# Cutaneous Neoplasms with Sebaceous Differentiation: A Comprehensive Update on Clinical, Histopathological and Molecular Classification

**DOI:** 10.3390/cancers18193209

**Published:** 2026-10-04

**Authors:** Katharina Wiedemeyer, Ingrid Ferreira, David J. Adams, Thomas Brenn

**Affiliations:** 1 Department of Pathology, University of Michigan, Ann Arbor, MI 48109, USA; 2 Department of Pathology, Clinique Universitaire Saint-Luc, Université Catholique de Louvain (UCLouvain), 1200 Brussels, Belgium; 3 Experimental Medicine Unit, de Duve Institute, Université Catholique de Louvain (UCLouvain), 1200 Brussels, Belgium; 4 Somatic Functional Genomics and Cancer, Wellcome Sanger Institute, Wellcome Genome Campus, Hinxton, Cambridge CB10 1SA, UK

**Keywords:** sebaceous adenoma, sebaceoma, sebaceous carcinoma, Muir–Torre syndrome, Lynch syndrome, skin adnexal tumours with sebaceous differentiation, nevus sebaceus

## Abstract

Cutaneous sebaceous neoplasms are rare adnexal tumours differentiated towards sebaceous glands, spanning a biological spectrum from benign (sebaceous adenoma, sebaceoma, nevus sebaceus) to outright malignant (sebaceous carcinoma). Most tumours are sporadic and solitary, with a predilection for the head and neck of elderly adults, but a clinically important subset is observed in the setting of Lynch syndrome/Muir–Torre syndrome. Recent large-scale genomic and transcriptomic studies have provided insights into the pathogenesis of these unique neoplasms, revealing a genetically heterogeneous landscape shaped by mismatch repair deficiency, UV damage, and abnormalities in several other key signalling pathways. The inclusion of nevus sebaceus within the sebaceous tumour family in the WHO 5th Edition of Skin Tumours reflects a conceptual shift, recognising nevus sebaceus as a clonal, mutation-driven neoplasm and a precursor lesion for secondary tumour development.

## Simple Summary

Cutaneous sebaceous neoplasms are rare skin adnexal tumours with differentiation towards sebaceous glands. They range from benign lesions, such as sebaceous adenoma and nevus sebaceus, to sebaceous carcinoma. Most are sporadic and occur on the head and neck of older adults. However, some are associated with Lynch syndrome/Muir–Torre syndrome. Therefore, accurate diagnosis of tumours with sebaceous differentiation is clinically important because they can serve as early markers of Lynch syndrome. Recent studies show a diverse genetic landscape, including mismatch repair deficiency, UV-related damage, human papilloma virus infection and alterations in key signalling pathways.

## 1. Introduction

Cutaneous neoplasms with sebaceous differentiation are classified under the “Sebaceous tumours” family within the appendageal tumour category according to the World Health Organization (WHO) Classification of Skin Tumours, 5th Edition [1]. The recognised entities include sebaceous adenoma, sebaceoma, sebaceous carcinoma, and nevus sebaceus. While traditionally regarded as a hamartoma, nevus sebaceus has been included in the sebaceous tumour family in view of the underlying genetic alteration of *RAS* genes in both nevus sebaceus and benign sebaceous tumours [2,3]. In contrast, sebaceous hyperplasia is not recognised as a tumour entity, primarily because it has traditionally been considered a reactive or physiological process—an increase in the number of normal-appearing sebaceous lobules without the architectural or cytological abnormalities that define a neoplasm [4]. Some authors have challenged this distinction [5].

## 2. Sebaceous Adenoma

### 2.1. Epidemiologic and Clinical Data

Sebaceous adenoma is a rare, benign neoplasm that predominantly affects middle-aged and older adults, with a mean age of approximately 69 years [6,7]. Precise population-based incidence and prevalence data for sebaceous adenoma are limited, reflecting its rarity [2,8,9,10]. The vast majority arise on the head and neck, particularly the nose, cheeks, face, scalp, and eyelids. The sex distribution is roughly even, with a slight male predominance in some series. Most patients (83.3%) [2] present with a solitary, skin-coloured, yellow or tan papule, typically about 5 mm in greatest dimension, although it may reach several centimetres in size [7,11]. Occasional ulceration may occur. Sebaceous adenoma is frequently mistaken for basal cell carcinoma on clinical examination [4]. The development of more than one sebaceous tumour and occurrence on a location outside the head and neck, as well as onset of sebaceous tumours before 50 years of age, is strongly associated with Lynch syndrome/Muir–Torre syndrome (MTS) [12].

### 2.2. Histopathology

**Figure 1:**
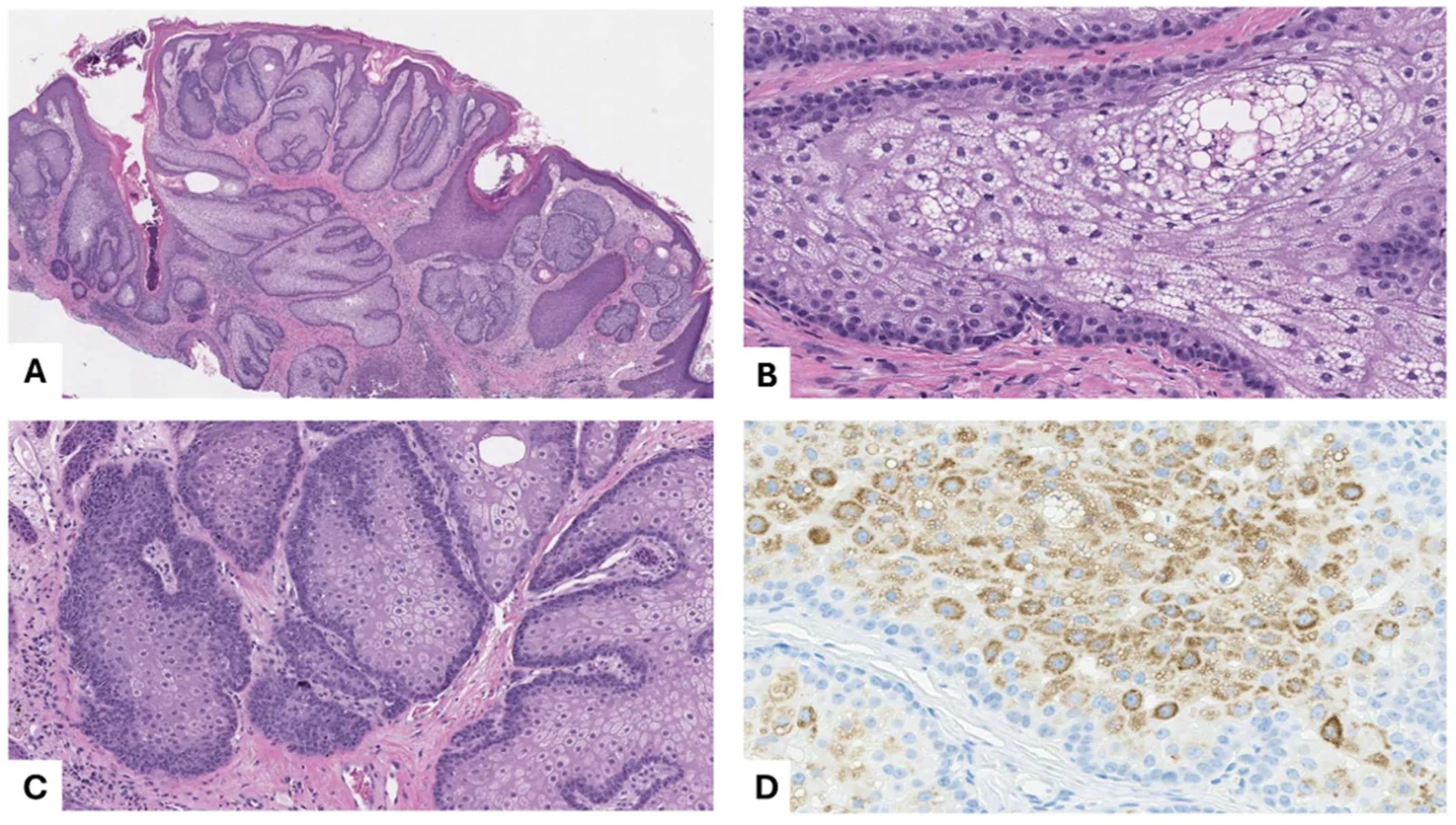
Sebaceous adenoma is a well-circumscribed, lobulated dermal neoplasm composed of central mature sebocytes and a peripherally expanded germinative basaloid cell layer (**A**–**C**). The tumour shows connection to the overlying epidermis. Cytological atypia is absent and the mitotic activity is low. Adipophilin immunohistochemical stain highlights the lipid vacuoles within the cytoplasm of the sebocytes (**D**).

Sebaceous adenoma shows a well-circumscribed, lobulated architecture within the upper to mid-dermis, often connected to the overlying epidermis. The lobules are composed of central mature sebocytes with abundant pale, vacuolated cytoplasm and scalloped nuclei, surrounded by peripheral basaloid germinative cells that are expanded beyond the normal 1–2 cell layers; however, by convention, the germinative (basaloid) cell population comprises less than 50% of all tumour cells. Sebaceous duct formation with holocrine secretion is usually seen. Sebaceous adenoma shows no to minimal cytologic atypia, low mitotic activity, and no necrosis (Figure 1).

### 2.3. Immunohistochemistry

Immunohistochemistry is not needed to confirm the diagnosis. The immunohistochemical profile of sebaceous adenoma is shared with sebaceoma (see below).

### 2.4. Genetics

There is no single main genetic alteration unique to sebaceous adenoma, and no adenoma-specific driver mutation or fusion has been identified to date. The most prominent genetic feature of sebaceous adenoma is its association with mismatch repair (MMR) deficiency (sporadic or constitutional), which results in a high rate of insertions and deletions (indels) in dMMR (deficient in MMR) sebaceous adenomas. A recent study applied dNdScv, a statistical algorithm that infers positive selection from the ratio of non-synonymous to synonymous somatic mutations, to define drivers of disease biology and tumour evolution. In sebaceous adenoma, several drivers were identified, including *HRAS* (54.3%), *NOTCH1* (72.9%), *RREB1* (52.9%), *NOTCH2* (42.9%) and *TP53* (21.4%), alongside the key MMR gene *MSH2* (22.9%) [13]. Of these, somatic *MSH2* mutation was linked to germline pathogenic *MSH2* variants, presumably reflecting inactivation of the wild-type allele in variant carriers. *NOTCH2* mutation was also a feature of tumours from germline pathogenic MMR variant carriers. *NOTCH1*, *RREB1*, *HRAS*, and *TP53* were the four most frequently mutated drivers across all benign and malignant tumours [13].

### 2.5. Treatment

Management is typically complete surgical excision, which is curative, with recurrence being uncommon [14].

## 3. Sebaceoma

### 3.1. Epidemiologic and Clinical Data

Sebaceoma is an uncommon benign tumour with limited population-level incidence data. The term “sebaceoma” was introduced in 1984 to replace the older term “sebaceous epithelioma” and to distinguish it from sebaceous adenoma [15]. Subsequent studies debated whether sebaceous adenoma and sebaceoma are separate entities or part of a broader spectrum of benign sebaceous tumours [16]. Sebaceoma remains a distinct entity in the current WHO Classification of Skin Tumours. It is classified as a separate benign sebaceous neoplasm alongside sebaceous adenoma, with sebaceous carcinoma as the malignant counterpart.

Sebaceoma most commonly affects elderly patients, with a median age of 70 years in one large series, though reported ranges span from 22 to 89 years. There is a reported female predilection in some series, while other studies of organoid-patterned sebaceomas have shown a male-to-female ratio of 2:1. This discrepancy may reflect differences in the morphologic subtypes studied [17].

Clinically, sebaceomas appear as single skin-coloured or yellow nodules, typically 5–20 mm in size, though they may grow to several centimetres [4,15]. Sebaceoma may be associated with MTS/Lynch syndrome, and identification should prompt evaluation of personal and family history for Lynch syndrome criteria [18].

### 3.2. Histopathology

**Figure 2:**
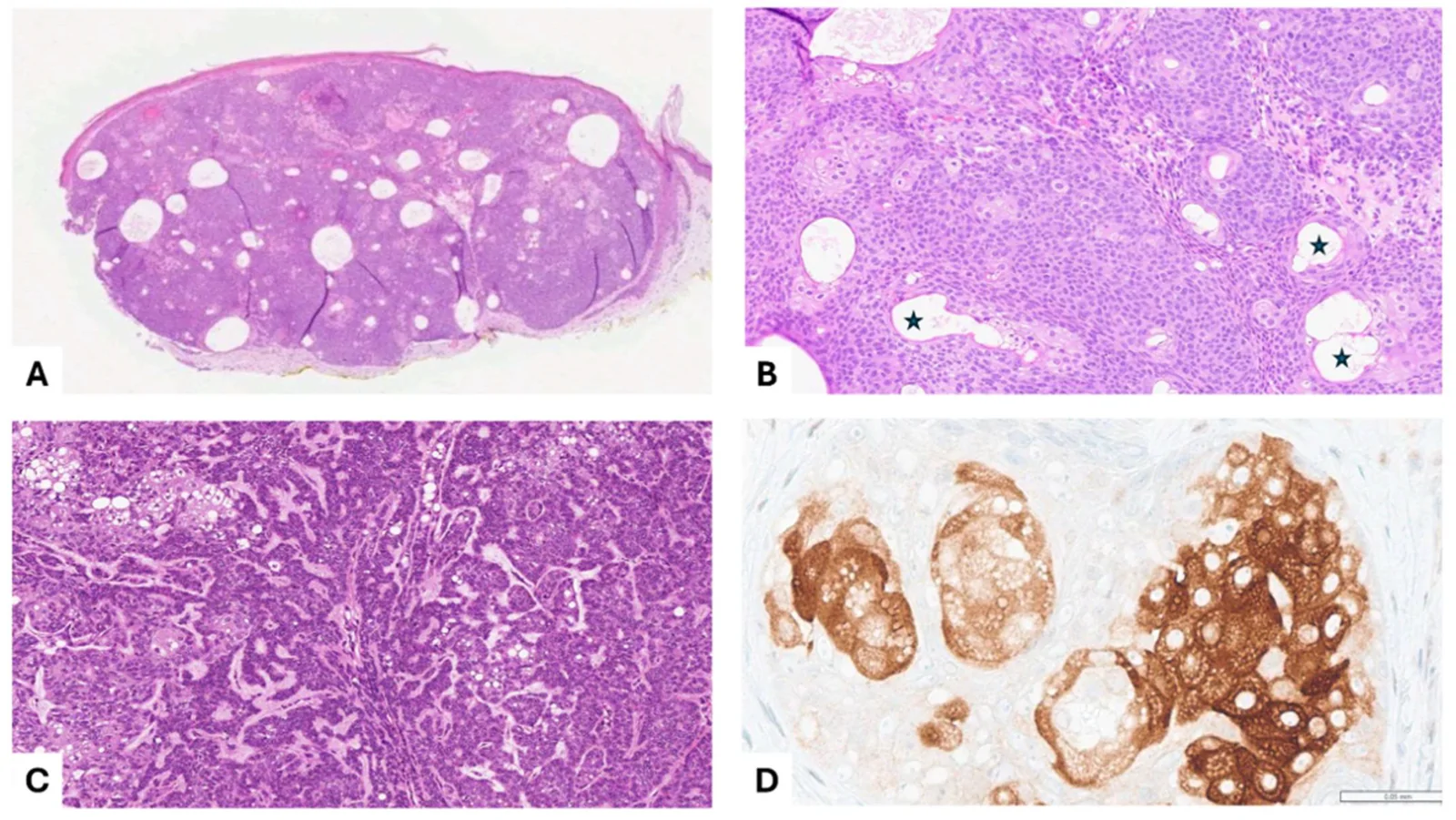
Sebaceoma is a sharply demarcated, uni- or multilobular dermal neoplasm composed predominantly (>50%) of immature basaloid germinative cells admixed with scattered mature sebocytes (**A**–**C**). Multiple sebaceous ducts are seen (**B**, marked by stars). Mitoses can be observed, but necrosis is absent. Sebaceomas can show an organoid growth pattern, including labyrinthine formations (**C**). The sebaceous differentiation is highlighted by cytoplasmic PRAME staining (**D**). Nuclei are spared by PRAME in sebaceous neoplasms.

Sebaceomas are sharply demarcated, uni- or multilobular dermal neoplasms with variable epidermal connection. Per definition, they are composed of >50% immature basaloid/germinative cells with scanty cytoplasm and vesicular nuclei, intermixed with various amounts of mature sebocytes [15,19] (Figure 2). There is a loss of the organoid maturation pattern, and the mature sebocytes appear haphazardly distributed rather than centrally maturing as in sebaceous adenoma. Sebaceous ducts and holocrine secretion are usually present (Figure 2B). Mitotic activity may be brisk, but atypical mitoses, nuclear pleomorphism, and necrosis are absent [7]. Distinctive architectural patterns include rippled, sinusoidal/labyrinthine (Figure 2C), and carcinoid-like patterns, found in approximately one-quarter of sebaceomas [17]. Infundibulocystic structures and squamous metaplasia may be present and can cause diagnostic confusion with trichoadenoma or microcystic adnexal carcinoma [20].

### 3.3. Immunohistochemistry

A practical immunohistochemistry (IHC) panel for supporting a diagnosis of sebaceous adenoma or sebaceoma includes EMA, androgen receptor, adipophilin, PRAME and BerEp4: EMA stains mature sebocytes, androgen receptor shows nuclear positivity, PRAME (Preferentially Expressed Antigen in Melanoma) shows cytoplasmic expression (Figure 2D) [21], adipophilin highlights lipid vacuoles of various amount in the cytoplasm (Figure 1D), and BerEp4 is usually negative [21,22,23]. The basaloid component in sebaceous neoplasms shows a weaker staining intensity for PRAME, and EMA and adipophilin may be very subtle or absent [24]. Sebaceomas are positive for cytokeratin 7, GATA3 and GATA6, while they are negative for cytokeratin 20 [25,26].

### 3.4. Genetics

The genetic landscape of sebaceoma (Table 1) is characterised by two broad categories: MMR deficiency (sporadic or constitutional) marked by high tumour mutational burden (TMB) with a high indel rate and specific driver genes, and MMR-proficient tumours harbouring recurrent point mutations or gene fusions. The molecular drivers of the majority of sebaceomas remain incompletely defined [27]. *HRAS* (50%), *NOTCH1* (51.4%)*, RREB1* (51.4%), NOTCH2 (34.3%) and *TP53* (32.9%) are the most important driver genes reported in sebaceoma with *NOTCH1*, *RREB1*, *NOTCH2* and *MSH2* more commonly related to dMMR tumours, and *ZNF750* and *TP53* linked to pMMR (proficient in MMR) tumours; *HRAS* is a driver in both. *TP53* is the fourth most mutated gene across all sebaceous neoplasms, although in benign tumours the mutations tend to be monoallelic (in contrast to biallelic *TP53* inactivation seen in carcinomas) [13,28]. *NOTCH1/2/3*—*NOTCH1* is the most frequently mutated gene overall in the largest molecular cartography study (286 tumours) [13]. In skin, *NOTCH1* functions as a tumour suppressor, and loss-of-function mutations disrupt sebocyte differentiation via the *JAG2/NOTCH1* signalling axis. *CDKN2A*, a cell-cycle regulator, is frequently mutated across benign and malignant sebaceous neoplasms. *KMT2D* is a chromatin remodelling gene that may be mutated in sebaceomas and sebaceous carcinomas, although it did not reach statistical significance in a large pan-sebaceous tumour study [13]. *TERT* promoter (*TERT*p) mutations are absent in benign sebaceous tumours [29]. A recent discovery is the identification of recurrent *GRHL* (Grainyhead-like) gene fusions in a molecularly distinct subset of sebaceomas. Across all 11 currently known *GRHL*-rearranged sebaceomas, these tumours are characterised by an organoid/macronodular architecture with cribriform growth, infundibulocystic structures, low mitotic activity, and MMR proficiency [13,27,30]. DNA methylation profiling shows that *GRHL*-fused sebaceomas form an independent epigenetic cluster, distinct from both conventional sebaceomas and *GRHL*-fused trichogerminomas, but with proximity to poromas with folliculo-sebaceous differentiation [27]. An additional recent interesting finding is the potential role of somatic mutations of *POLE* in sebaceoma, as well as *POLD1* in benign sebaceous tumours, both of which act as polymerase proof-readers [13].

### 3.5. Treatment and Prognosis

Sebaceomas are treated by conservative surgical excision. The practical recurrence and progression risk of a completely excised sebaceoma is negligible [31,32].

## 4. Sebaceous Carcinoma

### 4.1. Epidemiologic and Clinical Data

Sebaceous carcinoma is a rare, aggressive malignant neoplasm of the sebaceous glands, with an overall incidence of approximately 1–2 per million in the general population, and its incidence has been steadily increasing since the 1970s [33]. Men are affected at approximately twice the rate of women; however, periocular sebaceous carcinoma is more frequent in women, and extraocular tumours predominate in men. The incidence is highest in White patients (~2 per million), intermediate in Asian/Pacific Islanders (~1 per million), and lowest in Black patients (~0.5 per million). Periocular sebaceous carcinoma accounts for a disproportionately higher share of eyelid malignancies in India and East Asia (~30%) compared to Western countries (1.5–5%) [34]. Risk factors include immunosuppression (AIDS, solid organ transplant recipients, T-cell-depleting therapy), ultraviolet radiation, prior radiotherapy, including radiotherapy for retinoblastoma, and high-risk HPV infection [13,35,36]. Sebaceous carcinoma predominantly affects elderly patients, with a median age at diagnosis of 73–79 years.

The clinical presentation varies by anatomic location and can be divided into periocular and extraocular forms. Periocular tumours are located on the eyelids, with a strong predilection for the upper eyelid, supposedly due to the high density of meibomian glands, and present as slowly growing, pink or yellow nodules or eyelid thickening, frequently misdiagnosed as benign conditions such as chalazion or blepharoconjunctivitis [13,37]. Extraocular sebaceous carcinoma favours the head-and-neck area but can occur on the trunk, extremities, and genitalia. These tumours present as solitary, firm, erythematous nodules that are often ulcerated and may reach several centimetres in size [7]. Sebaceous carcinoma should prompt consideration of Muir–Torre/Lynch syndrome, although the strength of association varies importantly by anatomic site. The National Comprehensive Cancer Network (NCCN) Guidelines explicitly classify sebaceous carcinomas as Lynch syndrome-related neoplasms and recommend tumour screening for deficient mismatch repair (dMMR) on all sebaceous neoplasms, including sebaceous carcinomas, regardless of age at diagnosis [38].

### 4.2. Histopathology

**Figure 3:**
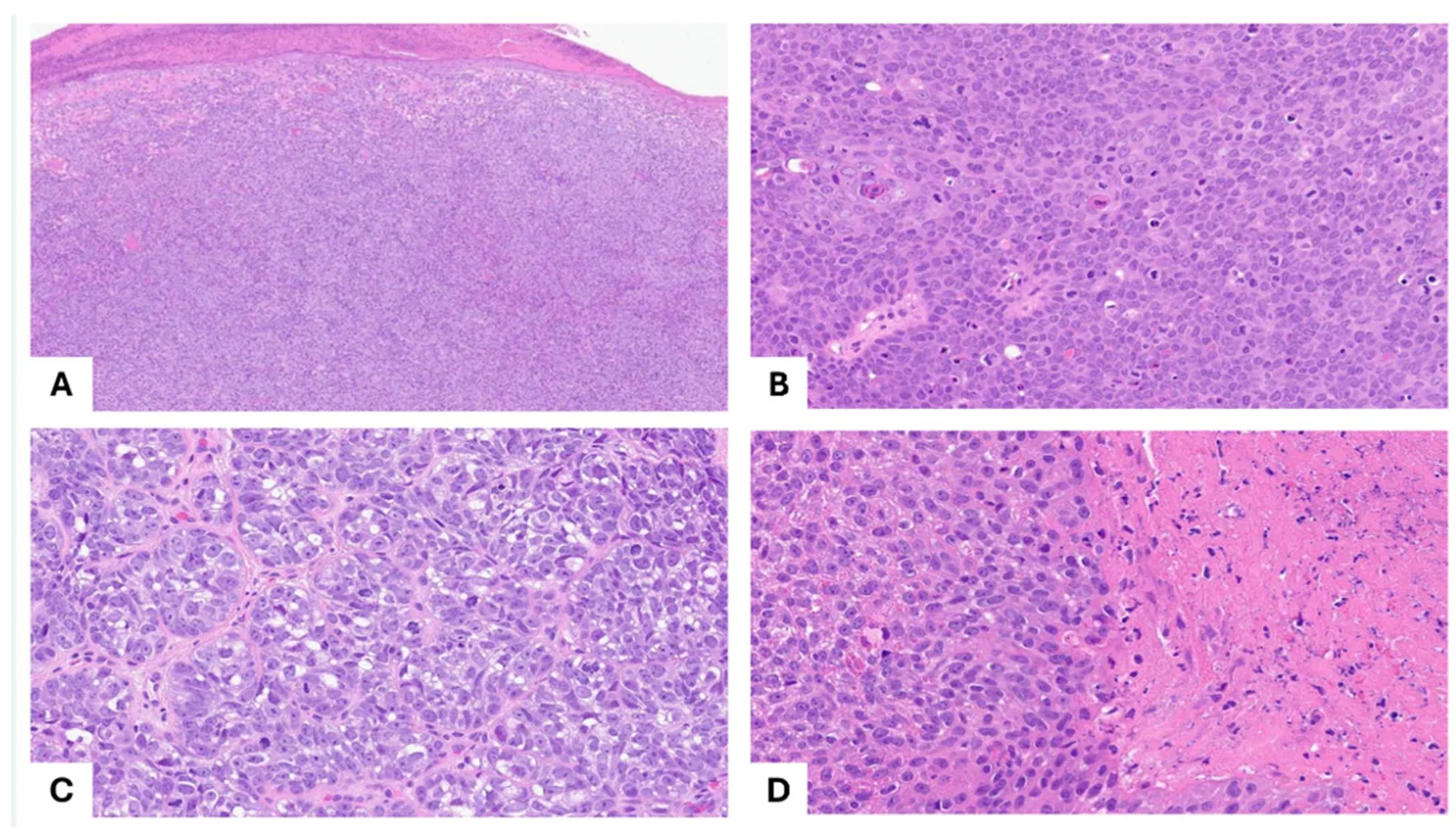
Sebaceous carcinoma may be erosive or ulcerated and show ill-defined, multinodular, diffusely infiltrative growth in the dermis and subcutaneous tissue (**A**). The tumour is mainly composed of basaloid polygonal cells with large irregular vesicular nuclei and prominent nucleoli but admixed with cells showing obvious sebaceous differentiation with vacuolated/clear cytoplasm and atypical, scalloped nuclei (**B**,**C**). The mitotic activity is high, and atypical mitoses are easily identified (**B**). Necrosis is present (**D**).

**Figure 4:**
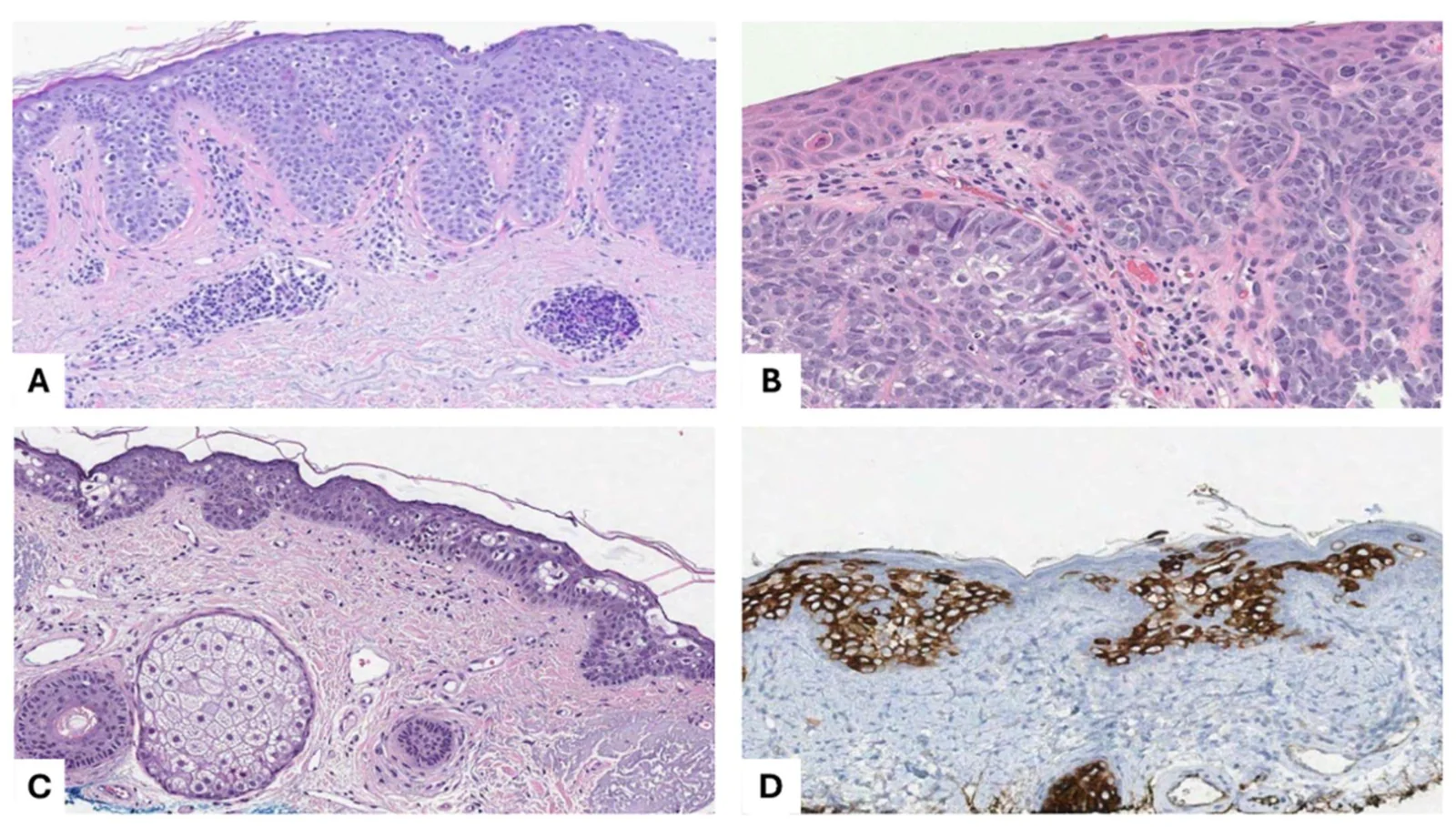
Sebaceous carcinoma in situ can appear very subtle and mimic high-grade squamous intraepithelial lesion/squamous cell carcinoma in situ (**A**–**C**). Especially when present in the genital area, the diagnosis is easily missed. Sebaceous carcinoma in situ and intraepithelial spread may be present at the periphery of invasive sebaceous carcinoma. Immunohistochemistry with adipophilin, androgen receptor and PRAME (**D**) is helpful to confirm the diagnosis.

Histopathologically, sebaceous carcinoma is usually an ill-defined neoplasm with a nodular, lobular, or diffusely infiltrative architecture extending into the dermis and subcutaneous tissue [39] (Figure 3). Circumscription and pushing borders are uncommon but have been described, and an epidermal connection may be present. Periocular tumours and a subset of tumours occurring on genital areas may show characteristic pagetoid intraepithelial spread with single cells or small nests replacing the epidermis [36,40] (Figure 4). The tumour is composed of atypical basaloid cells with limited cytoplasm admixed with variably differentiated sebocytes and foamy cytoplasm, including mature sebaceous cells with pale vacuolated cytoplasm, intracytoplasmic lipid vacuoles, and scalloped nuclei [28] (Figure 3). Cytologically, the neoplastic cells demonstrate large irregular vesicular nuclei, prominent nucleoli, granular or smudgy chromatin, and variable pleomorphism. Poorly differentiated tumours can show sheet-like basaloid proliferation, in which sebaceous differentiation may be subtle (Figure 3). Lymphovascular and perineural invasion in sebaceous carcinoma is uncommon, being reported in approximately 5–20% of cases, although exact prevalence data are limited due to the rarity of the tumour [41]. Mitotic activity is often brisk, frequently with atypical mitoses (Figure 3B), and tumour necrosis is common (Figure 3D), classically including comedo-type necrosis [42].

### 4.3. Immunohistochemistry

Sebaceous carcinoma shares the usual immunohistochemical profile of sebaceous differentiation (CK7+, EMA+, androgen receptor+, adipophilin+, PRAME+, BerEp4−) with sebaceous adenoma and sebaceoma. However, the staining may be more subtle or absent for CK7, PRAME, EMA and adipophilin in a subset of tumours or in poorly differentiated tumours [43]. Tumour cells with scant amounts of cytoplasm can be completely negative for all immunohistochemical markers but stain for bcl2 in a subset of tumours. Tumour cells that show minimal lipid vacuoles will only show a dot-like staining pattern for adipophilin. BerEp4 may show patchy positivity in a subset of tumours, but strong diffuse staining for BerEp4 should rase concern for basal cell carcinoma. P53 may show a wild-type staining pattern or overexpression [44]. The Ki-67 proliferative index is usually significantly higher in sebaceous carcinoma (~25–30%) than in sebaceoma (~10%) [26].

### 4.4. Genetics

Sebaceous carcinomas are characterised by (i) biallelic inactivation of the driver gene *TP53* co-occurring with *ZNF750* (also defined as a driver) and/or *RB1* mutations, except in (ii) HPV-driven carcinoma where *TP53*/*RB1* are wild-type [13,45], and (iii) chromosomal instability. These copy-number aberrations encompass significant amplification in 1q21.3 and chr20, and deletion of 13q14.3 (where *RB1* resides) [13]. Periocular sebaceous carcinomas are defined by a pauci-mutated profile with a low TMB with a low indel rate, no associated mutational signatures, and amplification of 8q12.1 (*LYN*, *PLAG1*), 20q13.33 (*GNAS*, *SALL4*), and 8q (*MYC*) [13,46]. In contrast, several sub-groups have been suggested in extraocular sebaceous carcinoma, such as: (i) dMMR extraocular sebaceous carcinoma driven by *NOTCH1* and/or *DRD5* with a co-occurring *RREB1* mutation—a high TMB with a high indel rate and MMR mutational signatures; (ii) pMMR UV-damaged/*APOBEC* activated extraocular sebaceous carcinoma harbouring a high TMB with a low indel rate, a *NOTCH1* mutation (or not) and a mutational signature related to UV or *APOBEC* activation; and (iii) pMMR pauci-mutated extraocular sebaceous carcinoma showing low TMB with a low indel rate with *HRAS* mutation. The genetic alterations of sebaceous carcinoma are summarised in Table 1.

### 4.5. Treatment and Prognosis

The 5-year overall survival is approximately 78% for localised/regional disease and 50% for metastatic disease [47]. Regional or distant metastasis can occur in up to 14–25% of cases [48]. A recent multicentre German analysis of 213 cases found a median overall survival of 61.4 months, with age being the only independent predictor of outcome—no significant survival difference was observed between periocular and extraocular disease [49]. Male sex, Black race, and extraocular location have been associated with higher all-cause mortality in SEER (Surveillance, Epidemiology and End Results) analyses [33]. Complete surgical excision with margin control is the mainstay of treatment for localised sebaceous carcinoma. Both wide local excision (WLE) and Mohs micrographic surgery (MMS) or frozen-section–controlled excision are used. In a multicentre cohort of 360 eyelid tumours, MMS was associated with substantially lower local recurrence than WLE (15.7% vs. 39.6%; adjusted HR 0.42, 95% CI 0.24–0.73) but no significant difference in metastasis or tumour-related mortality. National database analyses of overall survival are conflicting, with one reporting a survival advantage for MMS and another finding equivalent outcomes. The optimal modality therefore remains debated. For ocular tumours, surgical excision with frozen-section margin control is standard. Adjuvant topical chemotherapy (e.g., mitomycin C) and cryotherapy are used for conjunctival/pagetoid involvement to permit globe salvage, although topical therapy alone frequently fails to control intraepithelial disease. Orbital exenteration remains necessary in tumours with orbital extension. The role of conjunctival map biopsy is unsettled: some groups recommend it for all periocular tumours to detect pagetoid spread and guide management, while others found no clear benefit from routine four-quadrant biopsies and favour targeted biopsy of clinically suspicious areas. Given locoregional or distant spread in up to 14–25% of patients, clinical and radiologic nodal assessment is warranted, particularly for eyelid tumours, which carry a higher nodal risk than extraocular head-and-neck tumours. SLNB remains controversial: it may be considered for higher-stage periocular disease (≥T2c, AJCC 8th edition), where positivity exceeds 15% in experienced centres and pooled conjunctival series report positive nodes in about 8.6%, whereas the very low nodal rate in extraocular head-and-neck disease (~0.9%) supports observation there. No prospective data establish a survival benefit for SLNB in sebaceous carcinoma. Radiotherapy is used as an adjuvant treatment for locally advanced (≥T3a) or high-risk tumours, after nodal dissection, and for palliation, and as a definitive treatment for small tumours or non-surgical candidates, with the caveat that local control is inferior to surgery for larger lesions. Unresectable or metastatic disease is managed multimodally with neck dissection, radiotherapy, and systemic therapy; chemotherapy evidence is limited to case reports using 5-FU or platinum-based regimens extrapolated from head-and-neck cancer. Because a subset of SCs are mismatch repair-deficient/MSI-high, immune-checkpoint inhibition is a treatment option, supported by the approval of pembrolizumab for MSI-H/dMMR solid tumours and by case reports of durable responses, including in a microsatellite-stable tumour. However, a recent series found pembrolizumab to be ineffective as a first-line therapy for advanced pagetoid disease, while suggesting a possible role in recurrent or metastatic disease, and response is not universal even among dMMR/MSI-H tumours. Prolonged, risk-adapted surveillance is recommended because recurrence and metastasis can occur years after treatment (metastasis is reported up to 5 years later), with more intensive follow-up for high-risk features (size > 10 mm, poor differentiation, pagetoid spread, multicentricity, lymphovascular or orbital invasion). Finally, optimal care of periocular, high-risk, node-positive, or metastatic disease is best delivered through multidisciplinary discussion involving dermatology/Mohs surgery, oculoplastic and head-and-neck surgery, radiation oncology, medical oncology, pathology, and genetics.

## 5. Muir–Torre/Lynch/Lynch-like Syndrome and Related Conditions

All sebaceous neoplasms should prompt consideration of Muir–Torre syndrome (MTS, a phenotypic variant of Lynch syndrome), characterised by mismatch repair deficiency, except for (peri)ocular sebaceous carcinoma. MTS is defined by the synchronous or metachronous occurrence of at least one sebaceous neoplasm (adenoma, sebaceoma, or extraocular sebaceous carcinoma) and at least one Lynch syndrome-related visceral malignancy [50,51]. MTS is caused by germline mutations in DNA mismatch repair (MMR) genes, with *MSH2* being the most frequently mutated gene in MTS (68–93% of cases), resulting in microsatellite instability (MSI). The association of sebaceous and visceral tumours can also occur in patients with an unexplained mismatch repair-deficient (“Lynch-like”) phenotype. Lynch-like syndrome (LLS) has traditionally been used to describe colorectal, and, more broadly, Lynch-spectrum tumours, showing microsatellite instability (MSI) and/or loss of MMR protein expression by immunohistochemistry (IHC), in the absence of a detectable pathogenic germline *MMR*/*EPCAM* variant and without evidence of a sporadic cause such as somatic *MLH1* promoter hypermethylation (or, in appropriate colorectal cancers, BRAF V600E). LLS is a heterogeneous category rather than a single inherited syndrome: many cases are explained by biallelic somatic MMR alterations, while a minority may reflect other inherited cancer-predisposition mechanisms. Biallelic germline *MUTYH* variants, for example, have been identified in a small proportion of patients classified as having LLS (PMID: 24953332). LLS may be characterised by later-onset cancers and a less pronounced family history. MTS is present in approximately 28% of Lynch syndrome families and 9.2% of individuals with Lynch syndrome [52]. The mean age of presentation of sebaceous lesions is 53 years (range 23–89 years), and the median age for detection of the initial visceral neoplasm is 50 years (range 23–81 years) [53]. Associated visceral malignancies include colorectal, genitourinary, endometrial, gastric, pancreas, hepato-biliary, prostate, small bowel and ovarian carcinomas, and central nervous system glioblastoma. Sebaceous neoplasms precede or occur concurrently with the diagnosis of the initial visceral cancer in 9–52% of patients in LS patients [13,54].

### 5.1. Associated Sebaceous Neoplasms

**Figure 5:**
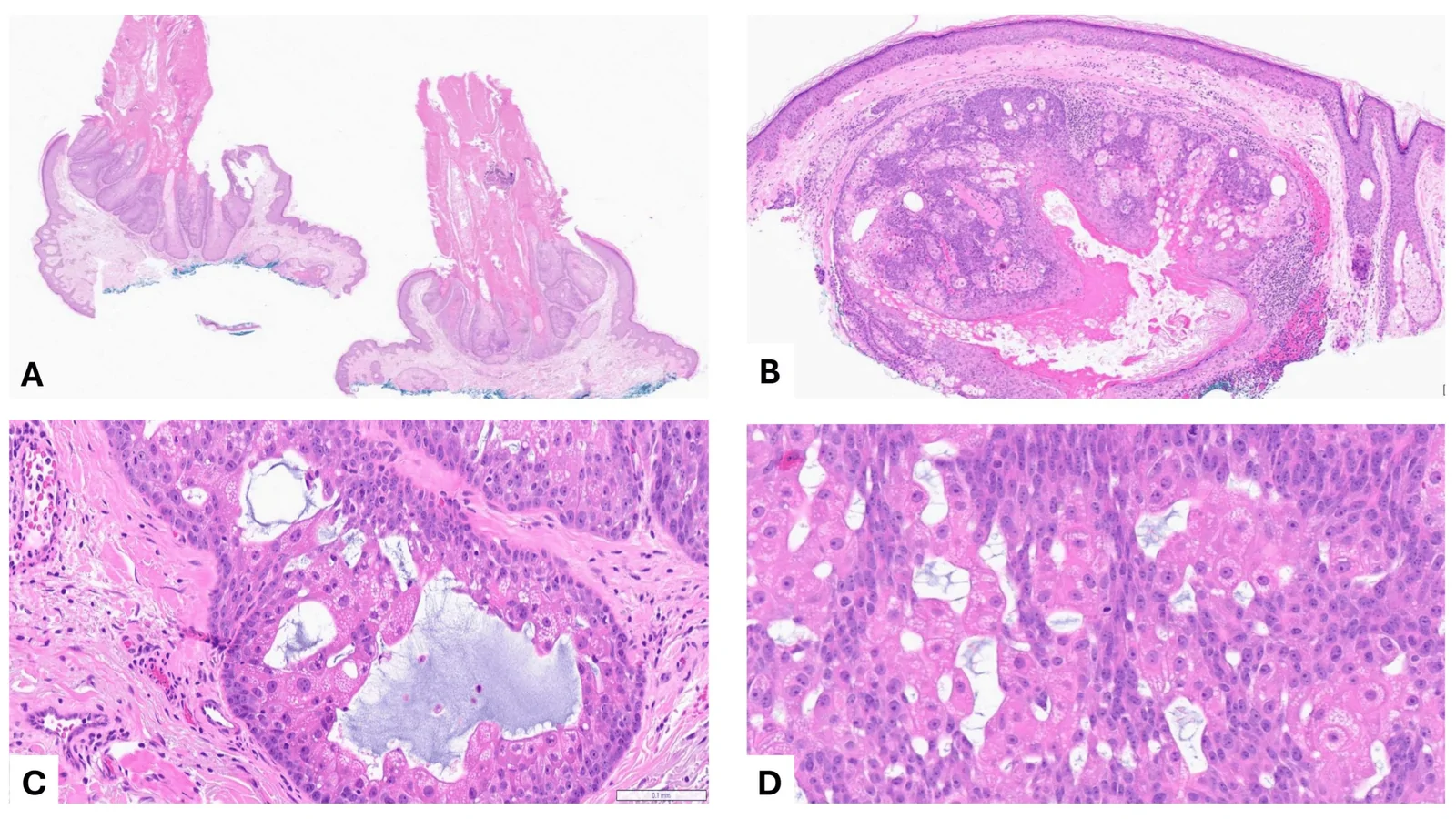
Sebaceous neoplasms associated with Lynch syndrome/MTS often show a keratoacanthoma-like architecture (**A**), cystic configuration (**B**) and mucinous areas (**C**,**D**). All tumours shown are sebaceous adenomas. The keratoacanthoma-like sebaceous neoplasms may easily be mistaken for conventional keratoacanthoma/well-differentiated squamous cell carcinoma. MMR deficiency may affect all cells of the pilosebaceous unit, and this shared origin provides a biological rationale for why the morphologic boundary between sebaceous neoplasms with keratoacanthoma-like features and conventional keratoacanthoma is particularly indistinct in MTS.

**Figure 6:**
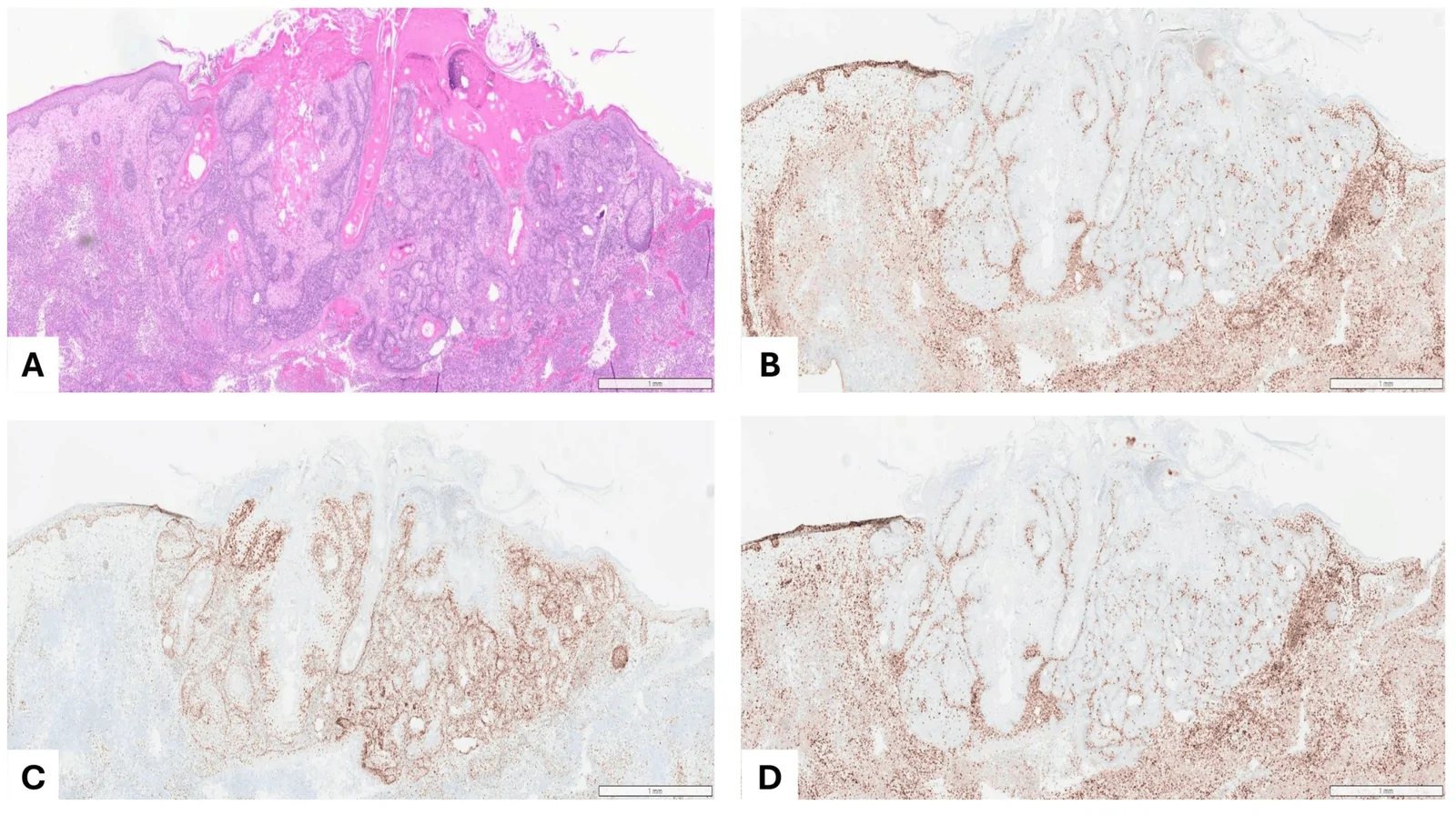
Sebaceous adenoma in a patient with Lynch syndrome/MTS: the tumour shows numerous intratumoural lymphocytes (**A**). Immunohistochemistry for mismatch repair proteins reveals loss of MSH2 (**B**) and MSH6 (**D**), while MLH1 and PMS2 are retained (MLH1 shown, **C**). Note that the intra-tumoural lymphocytes and peritumoural cells retain MMR proteins (**B**–**D**).

The most common cutaneous manifestation is sebaceous adenoma (~67% of patients). Sebaceoma and sebaceous carcinoma have also been reported in MTS with sebaceous carcinomas following a less aggressive course than sporadic sebaceous carcinomas and sebaceoma suggested to be the most predictive for MTS [13]. Sebaceous hyperplasia, periocular sebaceous carcinoma and nevus sebaceus are not a defining feature of MTS. The occurrence of multiple sebaceous tumours, sebaceous tumours developing outside the head-and-neck area and developing another non-sebaceous Lynch syndrome-related neoplasm should raise strong suspicion for MTS. Morphological features suggestive of MMR deficiency include cystic change, keratoacanthoma-like architecture, intratumoural mucin deposits, squamous metaplasia, lymphocytic infiltrates, and intertumoural heterogeneity, no connection to the overlying epidermis and delimitation of the tumour by a collarette [13,31]. The histopathological features are illustrated in Figure 5 and Figure 6.

### 5.2. Immunohistochemistry

Immunohistochemistry of DNA mismatch repair proteins MLH1, MSH2, MSH6 and PMS2 (Figure 6B–D) should be considered on all sebaceous tumours, except for periocular sebaceous carcinoma. The loss of at least one of these proteins should raise suspicion for an underlying Lynch/Muir–Torre syndrome, which remains a clinical diagnosis. Of note, *BRAF p.V600E* mutation and *MLH1* promoter hypermethylation testing cannot be used to distinguish sporadic from MTS-associated sebaceous tumours—as they can in colorectal cancer—because *BRAF p.V600E* mutations are essentially absent in all sebaceous neoplasms (both sporadic and syndromic), and somatic *MLH1* promoter hypermethylation does not appear to be a major mechanism driving sporadic MMR deficiency in sebaceous tumours [55,56].

## 6. Nevus Sebaceus

### 6.1. Epidemiologic and Clinical Data

Nevus sebaceus (NS), also known as nevus sebaceus of Jadassohn, is a congenital cutaneous hamartoma with a prevalence of approximately 0.3% of newborns (range 0.1–0.3% of live births), making it one of the most common congenital skin lesions and by far the most prevalent entity within the sebaceous neoplasm family [3,57]. The true prevalence is likely underestimated, as many lesions are asymptomatic and patients may never present for evaluation—particularly those with small, inconspicuous lesions on the scalp. The male-to-female ratio is approximately 1.3:1, with a mean age at first surgery of 12.5 years [58]. The overwhelming majority of NS occur on the head-and-neck region, with the scalp being the single most common site (66.4%), followed by the face (26.1%) [58,59]. NS presents at birth as a smooth, flat or slightly raised plaque with oval or linear configuration and is typically pink/orange to skin-coloured. During puberty, NS undergoes rapid growth and morphological transformation, becoming thickened, verrucous (cerebriform), and sometimes pruritic and friable.

### 6.2. Histopathology

**Figure 7:**
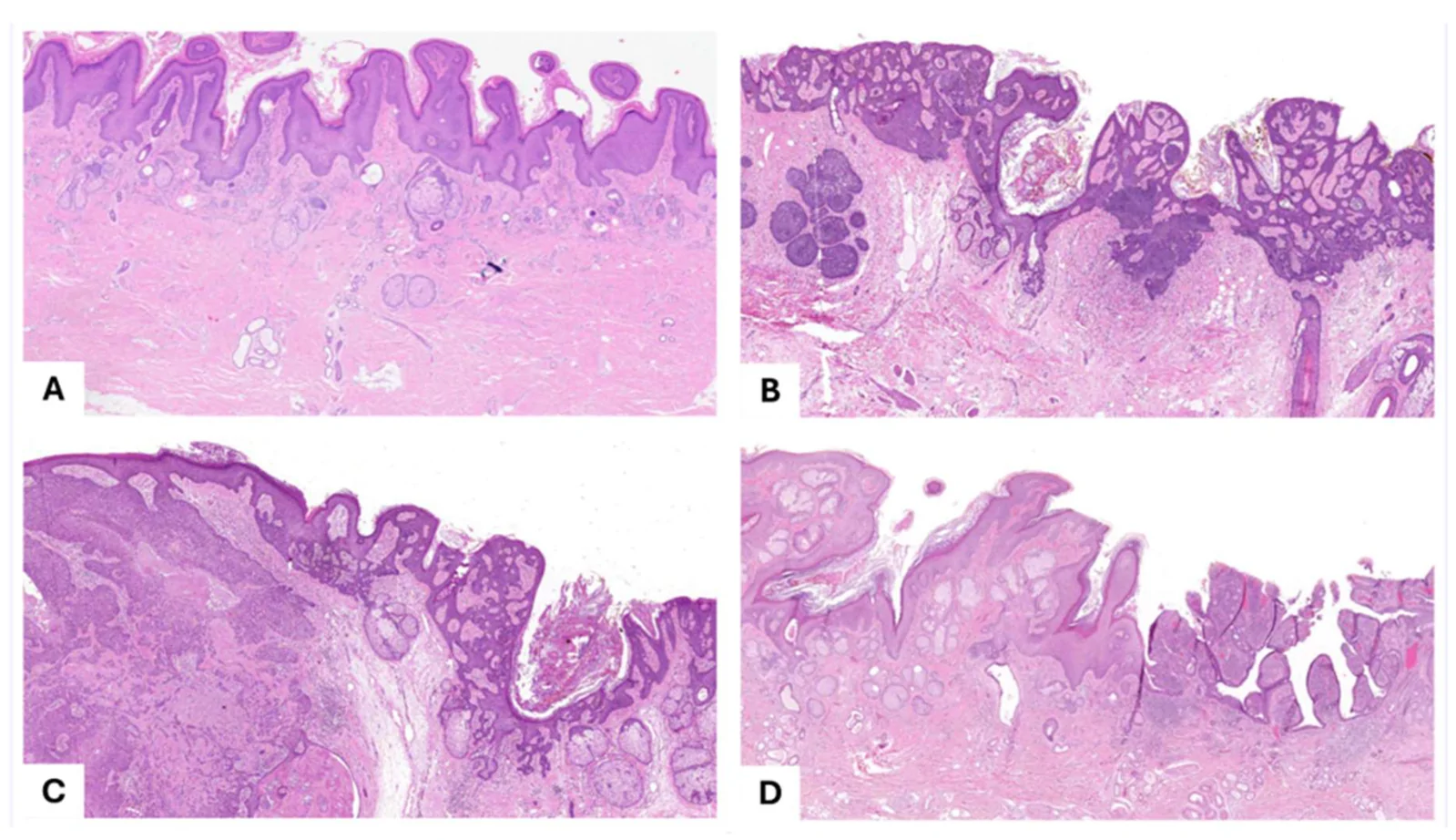
Nevus sebaceus (NS) presents with papillomatosis and increased sebaceous gland density, as well as enlarged apocrine glands in the dermis (**A**), and is typically located on the scalp. Secondary neoplasms, such as multifocal trichoblastoma (**B**), desmoplastic trichilemmoma (**C**) and syringocystadenoma papilliferum (**D**), may arise in NS.

In early stages, NS shows mild acanthosis, hyperkeratosis, immature pilo-sebaceous follicles, and underdeveloped sebaceous glands—making histological diagnosis challenging in some cases. During and after puberty, there is usually marked acanthosis with epidermal hyperplasia/papillomatosis, hyperplasia of sebaceous glands, and development of prominent apocrine glands (Figure 7A). The overall rate of secondary neoplasms within nevus sebaceus is approximately 12.8% [57]. Syringocystadenoma papilliferum and trichoblastoma (Figure 7B,D) are the most common secondary benign tumours arising in nevus sebaceus, followed by trichilemmoma [57,60] (Figure 7C). Trichoblastoma shows a striking female predominance and was historically misdiagnosed as basal cell carcinoma, inflating older estimates of malignant transformation rates in NS [61]. Other secondary benign neoplasms include sebaceous adenoma, sebaceoma, apocrine cystadenoma, apocrine adenoma, pilomatricoma, trichoadenoma, tumour of the follicular infundibulum, inverted follicular keratosis, proliferating trichilemmal cyst, syringoma, eccrine poroma, eccrine adenoma, hidradenoma, cutaneous mixed tumour and piloleiomyoma [62]. Secondary malignant tumours include basal cell carcinoma—almost exclusively arising in adults (mean age ~39–52 years) and squamous cell carcinoma. Squamous cell carcinoma arising in nevus sebaceus in children is extremely rare, with only a handful of documented cases. Rare malignancies include sebaceous carcinoma, apocrine carcinoma, syringocystadenocarcinoma papilliferum, microcystic adnexal carcinoma, trichilemmal carcinoma, mucoepidermoid carcinoma, and malignant melanoma (exceedingly rare, with only three documented cases) [63].

### 6.3. Immunohistochemistry

Routine immunohistochemical work-up is not needed for the diagnosis of NS. With adequate clinical history and an excisional specimen, the diagnosis is usually clear on H&E alone, based on the characteristic constellation of papillomatous epidermal hyperplasia, malformed sebaceous lobules, primitive follicular structures, reduced terminal follicles, and ectopic apocrine glands [64]. IHC might play a role in classifying secondary neoplasms arising in NS, which is discussed in the Differential Diagnoses section below.

### 6.4. Genetics

Nevus sebaceus is caused by postzygotic activating mutations in *RAS* pathway genes, most commonly *HRAS* (~95% of cases) and, less frequently, *KRAS* (~5%). sue [65] (Table 1). Nevus sebaceus is classified as a mosaic *RAS*opathy [3], caused by spontaneous, non-inherited genetic changes during embryonic development, meaning that only certain cell populations carry the mutation. Secondary neoplasms such as trichoblastoma and syringocystadenoma papilliferum arise in 10–30% of nevus sebaceus lesions and retain the underlying RAS mutation [66]. Whole-exome sequencing of secondary tumours has revealed additional putative driver mutations beyond the *RAS* alteration, including *TP53*, *MYCN*, *PTPN14*, *SMO*, and *NOTCH2* mutations, as well as copy-number loss of *PTCH1* [66,67]. Nevus sebaceus lesions without secondary tumours did not harbour these additional driver mutations or copy-number alterations, suggesting a multi-hit model for tumourigenesis within the nevus [66].

### 6.5. Treatment and Prognosis

The two main management approaches are clinical monitoring with selective excision versus prophylactic surgical excision, with the decision depending on patient age, lesion characteristics, cosmetic concerns, and risk tolerance. The overall prognosis is favourable, as the vast majority of secondary neoplasms are benign [59].

## 7. Differential Diagnosis of Sebaceous Neoplasms

The histopathological differential diagnosis of cutaneous sebaceous tumours spans a spectrum from benign to malignant entities, with important site-specific considerations—particularly the distinction between periocular and extraocular locations. Basal cell carcinoma—one of the most common cutaneous malignancies on the eyelid and on all other sites—is distinguished by peripheral palisading, retraction artefacts, strong and diffuse positivity of the tumour cells for BerEP4 and negativity for EMA, adipophilin and androgen receptor [44]. Basaloid or clear cell squamous cell carcinoma (SCC) can both mimic sebaceous carcinoma but are negative for adipophilin and androgen receptor and show no or weak expression of cytokeratin 7 [44]. Of note, squamous cell carcinoma and sebaceous carcinoma are positive for p63, p40 and EMA. Endocrine mucin-producing sweat gland carcinoma is an important differential diagnosis on the eyelid: it is a well-circumscribed tumour with cribriform/papillary growth patterns and intracellular mucin, but very mild atypia. It expresses androgen receptor and is also positive for EMA and BerEp4, which might be a diagnostic pitfall, but it is easily distinguished from sebaceous tumours by its positivity for neuroendocrine markers (INSM1, chromogranin, synaptophysin), low-molecular-weight keratins such as CAM5.2, and expression of oestrogen and progesterone receptors [68,69,70]. Other basaloid tumours with sweat gland differentiation, especially poroma and hidradenoma, need to be distinguished by the identification of duct-like lumina lined by cuboidal cells with eosinophilic cuticles highlighted by EMA and CEA. Poromas can show folliculo-sebaceous differentiation (“apocrine poroma”), which typically harbour a molecularly distinct set of alterations—recurrent PAK2 gene fusions (with various 5′ partners including RNF13, EPHB3, DLG1, LRIG1, ATP1B3, TM9SF4, and CTNNA1) [71]. Conventional (YAP1-fused) poromas can be identified by two complementary IHC surrogate markers: loss of YAP1 C-terminal expression and NUT (NUTM1) expression if needed. Hidradenoma may contain a various amount of clear cell differentiation which may mimic sebaceous differentiation, but the cytoplasm is glycogen-rich (PAS+, diastase-sensitive) and no intracytoplasmic lipids are seen (adipophilin and androgen receptor are negative). Hidradenoma often harbour MAML2 fusions, which can be detected using dual-colour break-apart FISH probes flanking the MAML2 locus at 11q21. These probes detect MAML2 rearrangement irrespective of the fusion partner (CRTC1, CRTC3) [72]. LINC00473 RNA in situ hybridisation (ISH) has been proposed as a potential surrogate biomarker for the detection of MAML2 fusion [73]. GRHL-rearranged sebaceomas with infundibulocystic structures, particularly when combined with squamous metaplasia, can cause confusion with trichoadenoma [74] or even microcystic adnexal carcinoma [20]. Sebaceoma with mainly basaloid immature sebocytes is often mistaken for trichoblastoma, which shows an intimately associated dense, cellular fibroblastic stroma (highlighted by IHC markers CD10 and CD34) and mesenchymal body formation. Trichoblastoma may contain focal sebocytes but sebaceous ducts are absent [19]. It is BerEp4-positive and shows increased Merkel cells within the overlying epidermis (highlighted by CK 20) [75] while being negative for EMA, androgen receptor, PRAME and adipophilin [24,76]. Sebaceous tumours arising in the genital area, particularly sebaceous carcinoma with pagetoid intraepithelial spread, need to be distinguished from extramammary Paget’s disease (EMPD) and high-grade squamous intraepithelial neoplasia (HSIL) and squamous cell carcinoma. EMPD shares a CK7+/CK20− immunophenotype with possible androgen receptor positivity and CEA positivity with sebaceous tumours, but it shows mucinous differentiation (mucicarmine+, PAS+, adipophilin−) and is positive for gross cystic disease fluid protein (GCDFP-15), and HER2 is positive in a subset of cases [77,78,79]. HSIL and squamous cell carcinoma in situ are particularly challenging to differentiate from intraepithelial sebaceous carcinoma, with adipophilin (positive in sebaceous carcinoma, negative in HSIL/SCC in situ) being the single most discriminating marker [23]. HSIL and sebaceous carcinoma with pagetoid spread can occur simultaneously, especially in immunocompromised patients with high-risk HPV infection [36]. Poorly differentiated sebaceous carcinoma may mimic Merkel cell carcinoma, which is distinguished by expression of perinuclear dot-like keratin and neuroendocrine markers, including INSM1, chromogranin, and synaptophysin [80].

## 8. Conclusions

Although cutaneous sebaceous neoplasms are increasingly recognised as a molecularly heterogeneous tumour family, their classification and diagnosis continue to rely primarily on histomorphology, and these molecular advances have not yet led to changes in established treatment strategies. The diagnostic, prognostic, and therapeutic significance of the emerging molecular landscape requires further validation through collaborative, multi-institutional studies, particularly given the rarity of these tumours. In addition, the morphological criteria distinguishing sebaceous adenoma from sebaceoma remain a matter of ongoing debate.

## Figures and Tables

**Table 1:** Overview of sebaceous neoplasms with their key molecular profiles and syndromic associations.

Neoplasm	Key Somatic Mutations	Copy-Number Alterations	Mutational Signatures/Other	Syndromic Associations
**Nevus sebaceus**	*HRAS*	NA	Postzygotic mosaic; constitutive MAPK and PI3K-AKT activation	Schimmelpenning syndrome
**Sebaceous adenoma**	*TP53* (D) *HRAS* (D) *NOTCH1* (D) *MSH2* (D) *RREB1* (D)			
dMMR	*HRAS* (D) *NOTCH1* (D) *MSH2* (D) *NOTCH2* (D) *RREB1* (D)	cnLOH of *MSH2*	high TMB and high indel rate microsatellite instability Mutation signatures: MMR (SBS15-21-26), MMR+*POLD1* (SBS20)	MMR genes (*MSH2* > *MLH1*, *MSH6*): Muir–Torre/Lynch syndrome; *MUTYH*: *MUTYH*-associated polyposis
**pMMR**	*HRAS* (D) *NOTCH1* (D) *RREB1* (D)	Minimal	low TMB and low indel rate Mutation signatures: UV (SBS7a-b-c) Fusion genes: *PAK2* fusions	
**Sebaceoma**	*HRAS* (D) *NOTCH1* (D) *RREB1* (D) *NOTCH2* (D) *TP53* (D) *ZNF750* (D) *MSH2* (D)			
**dMMR**	*NOTCH1* (D) *RREB1* (D) *HRAS* (D) *NOTCH2* (D) *MSH2* (D) *TP53* (D)	cnLOH of *MSH2*	high TMB and high indel rate microsatellite instability Mutation signatures: MMR (SBS15-21-26), MMR+*POLD1* (SBS20), *POLE* exonuclease proofreading defects (SBS10a-b)	MMR genes: Muir–Torre/Lynch syndrome; *MUTYH*-associated polyposis
**pMMR**	*HRAS* (D), *ZNF750* (D), *TP53* (D), *KRAS* (D)	Minimal	low TMB and low indel rate Mutation signatures: UV (SBS7a-b-c) Fusion genes: *GRHL1/GRHL2* fusions (*RCOR1::GRHL2*, *BCL6::GRHL1*, *BCOR::GRHL2*, *TLE1::GRHL1*) in organoid-patterned subset, *PAK2* fusions	
**Sebaceous** **carcinoma**	Biallelic inactivation of *TP53*, *TERTp* mutations	Chromosomal instability including - Gain of 1q21.3 (incl. *HRNR*), chr20 - Loss of RB1 (incl. deletion of 13q14.3 and 13q)		
**Extraocular**	*TP53* (D) *ZNF750* (D)	Loss of 13q14.3 (incl. *RB1*)		
- **dMMR**	*NOTCH1* (D) *RREB1* *HRAS* *TP53* *RB1* *DRD5* (D)	cnLOH of *MSH2*	high TMB and high indel rate Mutational signatures: MMR (SBS15-21-26) Histology: well-differentiated histology	MMR genes: Muir–Torre/Lynch syndrome
- **pMMR**	*ZNF750* (D) *TP53* (D) *NOTCH1* *RREB1* *HRAS* *RB1*			
- **UV-damage/APOBEC subtype**	*NOTCH1* *RREB1* *HRAS* *TP53* *ZNF750* *RB1*		high TMB and low indel rate; Mutational signatures: UV (SBS7a-b-c) and/or APOBEC activation (SBS2-13) Histology: poorly differentiated, infiltrative; squamous differentiation	
- **HPV-driven**	**no** *TP53* **no** *RB1*		low TMB and low indel rate Mutational signatures: none Clinic: occurs mostly in the genital area	
- **pauci-mutational subtype**	*HRAS* *NOTCH1* *TP53* *ZNF750* *RB1*		low TMB and low indel rate Clinic: occurs mostly on the face	
**Periocular**				
- **HPV-driven**	**no** *TP53* **no** *RB1* **no** *NOTCH1*			
- **pauci-mutational subtype**	*TP53* (D) *ZNF750* (D) *NOTCH1* (D) *RB1* *PCDH15* (related to metastasis) *PIK3CA*	- Gain of 8q12.1 (incl. *LYN*, *PLAG1*), 20q13.33 (incl. *GNAS*, *SALL4*), and 8q (incl. *MYC*) - Loss of 13q (incl. *RB1*)	low TMB and low indel rate Mutational signatures: No Clinic: older patients, frequent recurrence	Rarely hereditary retinoblastoma

Legend: APOBEC, apolipoprotein B mRNA editing enzyme, catalytic polypeptide-like; cnLOH, copy-neutral loss of heterozygosity; D, driver gene; dMMR, deficient in MMR; Mb, megabase; MMR, mismatch repair; pMMR, proficient in MMR; POLD1, DNA polymerase delta 1, catalytic subunit; POLE, DNA polymerase epsilon, catalytic subunit; SBS, single base substitution signature, TMB, tumour mutational burden; indel, insertions and deletions.

## Data Availability

No new data were created or analyzed in this study. Data sharing is not applicable to this article.
